# Mechanisms Underlying the Comorbidity of Schizophrenia and Type 2 Diabetes Mellitus

**DOI:** 10.1093/ijnp/pyaa097

**Published:** 2020-12-14

**Authors:** Yutaka Mizuki, Shinji Sakamoto, Yuko Okahisa, Yuji Yada, Nozomu Hashimoto, Manabu Takaki, Norihito Yamada

**Affiliations:** 1 Department of Neuropsychiatry, Okayama University Graduate School of Medicine, Dentistry and Pharmaceutical Sciences; 2 Shimonoseki Hospital; 3 Okayama Psychiatric Medical Center

**Keywords:** DISC1, kalirin, ARHGEF11, Akt/GSK3β, Wnt/β-catenin

## Abstract

The mortality rate of patients with schizophrenia is high, and life expectancy is shorter by 10 to 20 years. Metabolic abnormalities including type 2 diabetes mellitus (T2DM) are among the main reasons. The prevalence of T2DM in patients with schizophrenia may be epidemiologically frequent because antipsychotics induce weight gain as a side effect and the cognitive dysfunction of patients with schizophrenia relates to a disordered lifestyle, poor diet, and low socioeconomic status. Apart from these common risk factors and risk factors unique to schizophrenia, accumulating evidence suggests the existence of common susceptibility genes between schizophrenia and T2DM. Functional proteins translated from common genetic susceptibility genes are known to regulate neuronal development in the brain and insulin in the pancreas through several common cascades. In this review, we discuss common susceptibility genes, functional cascades, and the relationship between schizophrenia and T2DM. Many genetic and epidemiological studies have reliably associated the comorbidity of schizophrenia and T2DM, and it is probably safe to think that common cascades and mechanisms suspected from common genes’ functions are related to the onset of both schizophrenia and T2DM. On the other hand, even when genetic analyses are performed on a relatively large number of comorbid patients, the results are sometimes inconsistent, and susceptibility genes may carry only a low or moderate risk. We anticipate future directions in this field.

## Introduction

Schizophrenia is found in all cultures and appears to affect 0.5% to 1.5% of people during their lifetime (Pedersen et al., 2014). Due to its early age of onset and subsequent tendency to persist chronically, it produces great suffering for patients and their family members (Weinberger and Harrison, 2011). The mortality rate of patients with schizophrenia is twice as high as that of the general population, and their life expectancy is 10–20 years shorter (Crump et al., 2013; Lawrence et al., 2013). Although suicide and other unnatural causes account for more than 10% of the excess mortality, a substantial proportion of this excess mortality is due to the increased comorbidity of various medical illnesses in patients with schizophrenia (Crump et al., 2013; Lawrence et al., 2013; Olfson et al., 2015). Patients with schizophrenia have an increased risk for development of type 2 diabetes mellitus (T2DM). The prevalence of T2DM in patients with schizophrenia is approximately 6% to 21%, 2 to 3 times higher than in the general population (Mitchell et al., 2013; Stubbs et al., 2015). T2DM manifests as persistent hyperglycemia due to pancreatic beta-cell dysfunction, which leads to long-term complications. T2DM is a major risk factor for cardiovascular disease, and cardiovascular disease is the main cause of a substantial proportion of excess deaths of patients with schizophrenia (Murray et al., 2012; Lawrence et al., 2013; Olfson et al., 2015).

The mechanisms of the increasing prevalence of T2DM in patients with schizophrenia are multifactorial. T2DM and schizophrenia are caused by shared etiological factors (Ward and Druss, 2015). Traditional risk factors include a sedentary lifestyle and poor diet (Ward and Druss, 2015). Risk factors unique to schizophrenia include low socioeconomic status, cognitive dysfunction, and iatrogenic risk during treatment with antipsychotics (Ward and Druss, 2015). Some evidence suggests that a longer duration of schizophrenia increases the risk for diabetes (Philippe et al., 2005; Nuevo et al., 2011). Patients who have had schizophrenia for more than 25 years have nearly twice the risk for diabetes as those with less than 25 years since the first admission to hospital (Philippe et al., 2005). It has been found that impaired hormonal regulation of appetite, in terms of low leptin and high insulin levels, often occurs in early psychosis before antipsychotic treatment (Misiak et al., 2019; Lis et al., 2020a, 2020b). Hence, schizophrenia itself is a risk for increased onset of diabetes. Apart from these traditional risk factors and risk factors unique to schizophrenia, recent studies show an elevated risk of T2DM among drug-naïve or first-episode patients with schizophrenia and their relatives (Perry et al., 2016; Pillinger et al., 2017; Rajkumar et al., 2017). A recent systematic review and meta-analysis of glucose homeostasis in unaffected first-degree relatives of schizophrenia patients suggested impaired glucose tolerance in this population as well (Misiak et al., 2020). Thus, previous evidence has strongly suggested that schizophrenia and T2DM are caused by multiple genetic variants (Gough and O’Donovan, 2005). Multiple twin and family studies and heritability of intermediate phenotypes provide convincing evidence for an important role of the genetic etiologies, respectively (Das and Elbein, 2006; Demjaha et al., 2012). The risk of T2DM in patients with psychoses such as schizophrenia is elevated two- to fourfold in association with a positive family history of diabetes (Foley et al., 2014; Chung and Miller, 2020). Further, one-half of patients with schizophrenia are reported to have a family history of T2DM compared with 4.6% of healthy adult controls (Bushe and Holt, 2004). Interestingly, the polygenic risk score related to the onset of schizophrenia is also associated with insulin resistance in first-episode and antipsychotic-naive patients with schizophrenia (Tomasik et al., 2019). Thus, schizophrenia and T2DM may influence each other and share susceptibility gene variants.

Accumulating evidence indicates that potential environmental risk factors affecting both the premorbid phase and after the onset of schizophrenia include exposure to stress in early life, poor dietary habits, and a sedentary life style, as noted above. Stress leads to the alteration of several biological mechanisms that has been termed “allostasis” (Misiak et al., 2014). These processes enable adaptation to novel situations. However, their prolonged and cumulative activation exerts systemic and detrimental effects called the allostatic load (AL) (Juster et al., 2016; Misiak, 2019). The AL concept can be a useful framework for apprehending biological dysregulations related to chronic stress. Biological alterations associated with AL (AL mediators) in schizophrenia include a subclinical inflammatory state, enhanced oxidative stress levels, decreased level of neurotrophins, and impaired hypothalamic-pituitary-adrenal (HPA) axis response (Misiak et al., 2014). There are also markers that enable the measurement of AL (AL index). A higher AL index has been associated with a higher severity of positive and depressive symptoms, working memory impairments, lower general functioning, and health outcomes, including all-cause mortality (Misiak, 2019; Piotrowski et al., 2019).

In this review, we describe the comorbidity of schizophrenia and T2DM in genetic and functional pathways. First, we describe susceptibility genes common to schizophrenia and T2DM (Table 1). Second, we discuss molecular mechanisms that might explain a common functional cascade in schizophrenia and T2DM. Third, we describe common mechanisms in schizophrenia and T2DM, such as inflammation, oxidative stress, and HPA axis dysfunction.

**Table 1 1.** Common susceptibility genes in schizophrenia (SCZ) and type 2 diabetes mellitus (T2DM)

Candidate genes	Location	Official full name	Functions	Method	Samples	References
**Common cascade**						
*ACE* (Upstream of Akt/GSK3)	17q23.3	angiotensin I converting enzyme	Hydrolyze angiotensin I into angiotensin 2	GWAS	SCZ and T2DM database	Liu et al. (2013)
*ARHGEF11* (Rho GTPase)	1q23.1	Rho guanine nucleotide exchange factor 11	Numerous cellular process, synaptic plasticity	Association study	T2DM (*n* = 685) vs. Con (*n* = 461)	Böttcher et al. (2008)
				Association study	SCZ (*n* = 490) vs. Con (*n* = 500)	Mizuki et al. (2014)
*BCL9* (Wnt/β-catenin)	1q21.1	B-cell CLL/lymphoma 9	Wnt signaling pathway	Association study	SCZ (*n* = 4,187) vs. Con (*n* = 5,772)	Li et al. (2011)
				CNV analysis	Cumulative scoring evidence,based on 32 CNV studies in SCZ	Luo et al. (2014)
				GWAS	T2DM (*n* = 402) vs. Con (*n* = 1,092)	Anderson et al. (2015)
*COMT* (Upstream of Akt/GSK3)	22q11.21	catechol-O-methyltransferase	Degradation of catecholamines	CNV analysis	Cumulative scoring evidence,based on 32 CNV studies in SCZ	Luo et al. (2014)
				Association study	T2DM (*n* = 595) vs. Con (*n* = 725)	Xiu et al. (2015)
*TCF7L2* (Wnt/β-catenin)	10q25.2-3	transcription factor 7 like 2	Participates in the Wnt signaling pathway	Association study	T2DM (*n* = 2,201)	Lyssenko et al. (2008)
				GWAS	SCZ (*n* = 924), T2D (*n* = 822), comorbid (*n* = 505); controls (*n* = 1,125)	Hackinger et al. (2018)
**Inflammation**						
*ANXA1*	9q21.13	annexin A1	Anti-inflammatory activity, endogenous regulator of RhoA	GWAS	SCZ and T2DM database	Liu et al. (2013)
*APOE*	19q13.32	apolipoprotein E	Lipid homeostasis and inflammation	GWAS	SCZ and T2DM database	Liu et al. (2013)
*C4*	6p21.33	complement C4	Encodes complement factor 4, classical activation pathway	Association study	SCZ (*n* = 28,799) vs.. Con (*n* = 35,986)	Sekar et al. (2016)
*IL10*	1q32.1	interleukin 10	Cytokine produced by monocytes and lymphocytes	GWAS	SCZ and T2DM database	Liu et al. (2013)
**Inflammation & HPA axis**						
*IL1B*	2q14.1	interleukin 1 beta	Potent proinflammatory cytokine	Association study	T2DM (*n* = 200) vs. Con (*n* = 223)	Achyut et al. (2007)
				Association study	SCZ (*n* = 621) vs. Con (*n*= 531)	Kapelski et al. (2015)
				Meta-analysis	SCZ (*n* = 20,185) vs. Con (*n* = 20,542)	Hudson and Miller (2018)
*IL6*	7p15.3	interleukin 6	Inflammation and the maturation of B cells	Association study	T2DM Twins (*n* = 6,720)	Arora et al. (2011)
				Association study	SCZ (*n* = 621) vs. Con (*n*= 531)	Kapelski et al. (2015)
				Meta-analysis	SCZ (*n* = 19,022) vs. Con (*n* = 19,378)	Hudson and Miller (2018)
*TNF*	6p21.33	tumor necrosis factor	Encodes a multifunctional proinflammatory cytokine	GWAS	SCZ and T2DM database	Liu et al. (2013)
**Oxidative stress**						
*GSTM1*	1p13.3	glutathione S-transferase mu 1	Conjugattes reduced glutathione to hydrophobic electrophiles Detoxification of electrophilic compounds	Association study	SCZ (*n* = 111) vs. Con (*n* = 130)	Pae et al. (2004)
				Association study	SCZ (*n* = 138) vs. Con (*n* = 133)	Gravina et al. (2011)
				GWAS	SCZ and T2DM database	Liu et al. (2013)
				Meta-analysis	T2DM (*n* = 1,354) vs. Con (*n* = 1,666)	Tang et al. (2013)
				Meta-analysis	T2DM (*n* = 2,577) vs. Con (*n* = 4,572)	Zhang et al. (2013)
*GSTT1*	22q11.23	glutathione S-transferase theta 1	Conjugates reduced glutathione to hydrophobic electrophiles Detoxification of electrophilic compounds	Association study	SCZ (*n* = 138) vs. Con (*n* = 133)	Gravina et al. (2011)
				Meta-analysis	T2DM (*n* = 1,271) vs. Con (*n* = 1,470)	Tang et al. (2013)
				Meta-analysis	T2DM (*n* = 2,577) vs. Con (*n* = 4,572)	Zhang et al. (2013)
*MTHFR*	1p36.22	methylenetetrahydrofolate reductase	Catalyzes conversion of methylenetetrahydrofolate	GWAS	SCZ and T2DM database	Liu et al. (2013)
*PON1*	7q21.3	paraoxonase 1	Hydrolyzes toxic metabolites	GWAS	SCZ and T2DM database	Liu et al. (2013)
*SOD2*	6q25.3	superoxide dismutase 2	Destroys superoxide anion radicals	GWAS	SCZ and T2DM database	Liu et al. (2013)
TXNRD2	22q11.21	thioredoxin reductase 2	Regulation of mitochondrial redox homeostasis	CNV analysis	Cumulative scoring evidence,based on 32 CNV studies in SCZ	Luo et al. (2014)
				Association study	T2DM patients with myocardial infarction (*n* = 166) vs. Con (*n* = 811)	Kariž et al. (2015)
*UCP2*	11q13.4	uncoupling protein 2	Mitochondrial transporter protein	GWAS	SCZ and T2DM database	Liu et al. (2013)
**Prolactin pathway**						
*NPY*	7p15.3	neuropeptide Y	Control of feeding, secretion of gonadotrophin-release hormone	Association study	SCZ (*n* = 212) vs. Con (*n* = 199)	Itokawa et al. (2003)
				Association study	T2DM (*n* = 263) vs. Con (*n* = 469)	Nordman et al. (2005)
				GWAS	SCZ and T2DM database	Liu et al. (2013)
*PRL*	6p22.3	prolactin	Encodes anterior pituitary horomone prolactin	Association study	SCZ (*n* = 403) vs. Con (*n* = 653)	Rybakowski et al. (2012)
**Other**						
*DLGAP1*	18p11.31	DLG associated protein 1	Scaffold protein in postsynaptic density, glutamate neurotransmission	CNV analysis	SCZ (*n* = 662) vs. Con (*n* = 2,623)	Kirov et al. (2012)
				CNV analysis	T2DM (*n* = 1,715)	Prabhanjan et al. (2016)
*Herv K-18*	1q23.3	endogenous retrovirus group K member 18	CD48 signaling lymphocyte activating (SLAM) gene	Association study	SCZ with T2DM (*n* = 29) vs. SCZ (*n* = 200)	Dickerson et al. (2008)
*IGF2BP2*	3q27.2	insulin-like growth factor 2 mRNA binding protein 2	Embryonic growth and development, decrease insulin secretion	Association study	T2DM (*n* = 2,201)	Lyssenko et al. (2008)
				Association study	SCZ (*n* = 790) vs. Con (*n* = 1,083)	Zhang et al. (2013b)
*PACRG*	6q26	parkin coregulated	Molecular chaperone/chaperonin-binding	GWAS	SCZ (*n* = 924), T2D (*n* = 822), comorbid (*n* = 505); controls (*n* = 1,125)	Hackinger et al. (2018)
*PSMD9*	12q24.31	proteasome 26S subunit, non-ATPase 9	Chaperone of 26S proteasome complex assembly Insulin gene transcription coactivator	Association study	T2DM affected siblings/families (*n* = 201)	Gragnoli (2010)
				Association study	SCZ (*n* = 1,351) vs. Con (*n* = 1,378)	Lee et al. (2013)
*SRR*	17p13.3	serine racemase	Catalyzes synthesis of D-serine	GWAS	SCZ and T2DM database	Liu et al. (2013)
*SYN2*	3p25.2	synapsin Ⅱ	Synaptogenesis, modulation of neurotransmitter release	GWAS	SCZ and T2DM database	Liu et al. (2013)
*TSPAN18*	11p11.2	tetraspanin 18	Tetraspanin families which regulate cell development	GWAS	SCZ and T2DM database	Liu et al. (2013)

### Common Susceptibility Genes in Schizophrenia and T2DM

Previous family-based genome-wide linkage studies show that schizophrenia and T2DM have a number of overlapping risk loci, including chromosomes 1p13, 1p36, 1q21–24, 1q25, 2q14, 2q33, 2q36, 3p22, 3q29, 4q25, 5q13, 6p21, 6q25, 7p15, 7p21, 7q21, 7q31, and 9p24 (Lin and Shuldiner, 2010). These loci include gene-rich regions that will harbor multiple common candidate genes for susceptibility to schizophrenia and T2DM. Although many of these loci cover large distances in genomic DNA, chromosome 1q was reported to have a linkage to T2DM by several previous studies (Das and Elbein, 2007; Tziastoudi et al., 2019). This location has also been implicated as a schizophrenia susceptibility locus (Brzustowicz et al., 2000). Therefore, these findings have suggested that chromosome 1q may be remarkably rich in linkage findings for co-occurrence of schizophrenia and T2DM. Within this region of linkage, susceptibility genes for T2DM such as endogenous retrovirus group K member 18 (*Herv K-18*) and Rho guanine-nucleotide exchange factor 11 (*ARHGEF11*) (Böttcher et al., 2008) have been found to be associated with schizophrenia among sampled populations (Dickerson et al., 2008; Mizuki et al., 2014).

The most straightforward method to identify the genetic risk for comorbidity of schizophrenia and T2DM is searching for overlapped candidate genes or regions of these 2 individual diseases (Lin and Shuldiner, 2010). Common candidate genes shared by association studies focused on each individual disease are glutathione S-transferase mu 1 (*GSTM1*), glutathione S-transferase theta 1 (*GSTT1*), neuropeptide Y (*NPY*), and proteasome 26S subunit, non-ATPase 9 (*PSMD9*) (Itokawa et al., 2003; Pae et al., 2004; Nordman et al., 2005; Gragnoli, 2010; Lin and Shuldiner, 2010; Lee et al., 2013; Tang et al., 2013; Zhang et al., 2013a). According to data from the Genetic Association Database (http://geneticassociationdb.nih.gov/) (Becker et al., 2004), Catalog of Published Genome-Wide Association Studies (GWAS) (http://www.genome.gov/gwastudies/) (Hindorff et al., 2009), and Type 2 Diabetes Genetic Association Database (http://t2db.khu.ac.kr:8080/) (Lim et al., 2010), there are 196 schizophrenia susceptibility genes and 200 T2DM susceptibility genes. Among them, 14 genes (annexin A1 [*ANXA1*], apolipoprotein E [*APOE*], angiotensin I converting enzyme [*ACE*], *GSTM1*, interleukin 10 [*IL10*], methylenetetrahydrofolate reductase [*MTHFR*], *NPY*, paraoxonase 1 [*PON1*], superoxide dismutase 2 [*SOD2*], synapsin II [*SYN2*], tumor necrosis factor [*TNF*], uncoupling protein 2 [*UCP2*], serine racemase [*SRR*], and tetraspanin 18 [*TSPAN18*]) are common to both diseases (Liu et al., 2013). These genes could be divided into 2 functional categories. One category is inflammation-associated genes (*APOE*, *IL10*, *TNF*), and the other is genes that are involved in oxidative stress (*GSTM1*, *MTHFR*, *PON1*, *SOD2*, *UCP2*). Currently, the National Human Genome Research Institute–European Bioinformatics Institute catalog of published GWASs lists 402 schizophrenia-susceptibility genes and 890 genes associated with T2DM (Nagalski et al., 2016). They found 26 candidate genes that are shared by schizophrenia and T2DM. Functional analysis of the 79 candidate genes shared by T2DM and any of the severe mental illnesses (schizophrenia, bipolar disorder, and major depression) revealed several clusters of common candidate risk genes.

Other candidate genes, such as insulin like growth factor 2 mRNA binding protein 2 (*IGF2BP2*) or transcription factor 7 like 2 (*TCF7L2*), may also contribute to the genetic basis of the co-occurrence of schizophrenia and T2DM. The *IGF2BP2* polymorphisms are associated with vulnerability to schizophrenia in a Han Chinese population (Zhang et al., 2013b) and with impaired pancreatic β-cell function, including lower fasting insulin levels, which reduced glucose-stimulated insulin secretion (Lyssenko et al., 2008). The *TCF7L2* polymorphisms have been detected in consistent association with T2DM in multiple ethnic populations, including Japanese, Chinese, Americans, and Asian Indians (Wang et al., 2013). A GWAS in a Greek population identified genomic regions with evidence of colocalizing schizophrenia and T2DM. In this study, the most strongly associated variant resides within an intron of the Parkin coregulated (*PACRG*) gene in schizophrenia patients with T2DM vs controls, and another variant that reached genome-wide significance resides within the intron of the *TCF7L2* gene (Hackinger et al., 2018). However, the findings were negative in a GWAS performed in a Japanese population (Kajio et al., 2014).

Though common variants of SNP polymorphisms have only small effects, large, rare chromosomal copy number variants (CNVs) identified by comparative genomic analyses are known to increase the risk for schizophrenia and have relatively larger effects (Chen et al., 2015; Bray and O’Donovan, 2018). Of more than 20,000 schizophrenia patients and controls, 8 CNVs (on chromosomes 1q21.1, 2p16.3, 3q29, 7q11.2, 15q13.3, distal 16p11.2, proximal 16p11.2, and the velocardiofacial syndrome region on chromosome 22q11.2) are associated with the onset of schizophrenia at a significant genome-wide threshold (CNV and Schizophrenia Working Groups of the Psychiatric Genomics Consortium) (Marshall et al., 2017; Bray and O’Donovan, 2018). It is well known that 20%–30% of people with a 22q11.2 deletion have schizophrenia (Murphy et al., 1999), and the prevalence of obesity and T2DM is reported to be greater than normal in 22q11.2 deletion syndrome (Voll et al., 2017). A partial 22q11.2 deletion that includes several genes related to the neuropsychiatric phenotype, catechol-O-methyltransferase (*COMT*) (Xiu et al., 2015) and thioredoxin reductase 2 (*TXNRD2*) (Kariž et al., 2015), is associated with the onset of T2DM. Of 8 top candidate genes for schizophrenia affected by CNVs (Luo et al., 2014), only B-cell CLL/lymphoma 9 (*BCL9*) (1q21.1), which is required in the Wnt signaling pathway, was reported to be associated with T2DM (Anderson et al., 2015) or schizophrenia (Li et al., 2011), although there are not enough studies on the relationship between CNVs and T2DM, unlike schizophrenia. Prabhanjan et al. (2016) reported 24 CNV genes in patients with T2DM, and DLG associated protein 1 (*DLGAP1*), a scaffold protein of the postsynaptic density that is related to post-synapse neurotransmission of glutamate, is suspected to be genetically and functionally related to schizophrenia (Kirov et al. 2012; Rasmussen et al. 2017).

### Common Functional Cascade in Schizophrenia and T2DM

#### Rho GTPase—

Disturbances in synaptic connectivity during perinatal and adolescent periods underlie the pathophysiology of schizophrenia (McGlashan and Hoffman, 2000). Postmortem brain studies of individuals with schizophrenia have reported reduced dendritic spine density in the cerebral neocortex (Glantz and Lewis, 2000; Konopaske et al., 2014). These dendritic spine abnormalities are likely the result of disturbances in the molecular mechanisms that contribute to spine formation, pruning, and/or maintenance (Glausier and Lewis, 2013). Dendritic spine morphogenesis is regulated through cytoskeletal actin, which is concentrated highly in the spines (Fischer et al., 1998).

The Rho family of small GTPases (Rho GTPases), which includes Cdc42, Rac1, and RhoA, is a critical regulator of actin cytoskeleton dynamics and organization in the spines (Hall, 1998). The activation of Rho GTPases is mediated by speciﬁc guanine-nucleotide exchange factors (GEFs) that catalyze the exchange of bound GDP (inactive state) for bound GTP (active state) (Van Aelst and D’Souza-Schorey, 1997). Several Rho GEFs that localize to dendritic spines play important roles in dendritic spine morphogenesis by modulating the activity of Rho GTPases (Xie et al., 2007).

ARHGEF11 is a specific GEF for RhoA (Rumenapp et al., 1999). ARHGEF11 is expressed in the pancreas, liver, adipose tissue, and highly in the brain (Jackson et al., 2001). *ARHGEF11* variants are associated with a higher risk for the onset of schizophrenia in a Japanese population (Mizuki et al., 2014). ARHGEF11 interacts and colocalizes with synapse marker postsynaptic density protein 95 (PSD-95) at synapse sites and negatively regulates the formation of dendritic spines in cortical primary neurons (Mizuki et al., 2016). In a yeast 2-hybrid screen, ARHGEF11 interacts with disrupted-in-schizophrenia 1 (DISC1) (Millar et al., 2003). DISC1 directly interacts with PSD-95 and kalirin-7, a GEF for Rac1, and blocks access of kalirin-7 to Rac1. This binding is released by N-methyl-D-aspartate (NMDA) receptor activation, allowing free access of kalirin-7 to Rac1 and leading to the resultant activation of Rac1 and spine enlargement (Hayashi-Takagi et al., 2010). On the other hand, platelet-activating factor acetylhydrolase 1B1 (LIS1), one of the major binding partners of DISC1, is associated with RhoA activity (Kholmanskikh et al., 2003). Haploinsufficiency in LIS1 has also been shown to reduce spine density, while downregulation of RhoA rescued spine motility (Sudarov et al., 2013). DISC1 may also regulate the access of ARHGEF11 to RhoA, resulting in spine shrinkage. Regulation of Rho GTPases by *DISC1* may be crucial for proper maintenance of the dendritic spine (Tropea et al., 2018). *DISC1* (Ma et al., 2018; Xu et al., 2018) and *kalirin* (Kushima et al., 2012) are also reported to be associated with schizophrenia. Though *DISC1* is not considered a common risk gene for schizophrenia by GWAS, *DISC1* may play critical roles as a pathological mediator in a wide range of psychiatric disorders (Niwa et al., 2016).

**Figure 1.**
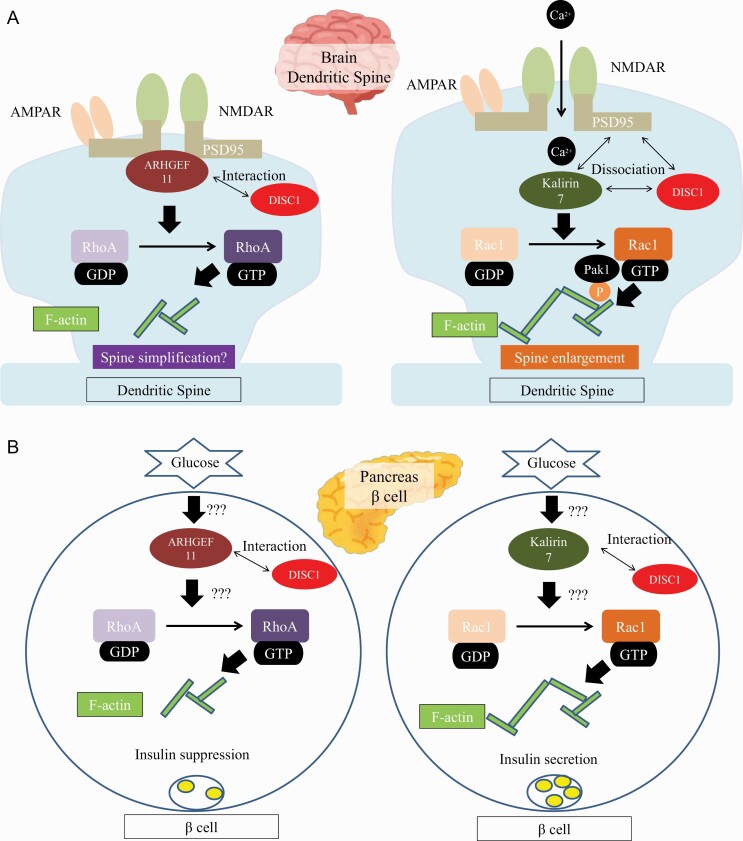
Summary of plausible shared mechanisms for the pathogenetic association between schizophrenia and type 2 diabetes mellitus (T2DM). (A) Schematic representation of Rho family of small GTPases (Rho GTPases) signaling cascades involved in synaptic plasticity. Rho guanine-nucleotide exchange factor 11 (*ARHGEF11*) interacts and colocalizes with synapse marker postsynaptic density protein 95 (PSD-95) at synapse sites and negatively regulated the formation of dendritic spines in cortical primary neurons (Mizuki et al., 2016). Disrupted-in-schizophrenia 1 (DISC1) directly interacts with PSD-95 and kalirin-7, a GEF for Rac1, and blocks access of kalirin-7 to Rac1. This binding is released by N-methyl-D-aspartate (NMDA) receptor activation, allowing free access of kalirin-7 to Rac1 and leading to the resultant activation of Rac1 and spine enlargement (Hayashi-Takagi et al., 2010). (B) The role of Rho GTPase in pancreatic β cells. Rac1 is particularly important for glucose-stimulated insulin secretion (Wang and Thurmond, 2009). In contrast, RhoA expression is increased in β-cells under diabetic conditions, and Rho/Rho-kinase activation is involved in the suppression of insulin biosynthesis (Nakamura et al., 2006). Insulin release from pancreatic islet β-cells could be determined by the resulting balance of Rho GTPase signaling. Illustrating this schematic figure, we referenced figures of Hayashi-Takagi et al., 2010 and Wang et al., 2009.

Dysfunction of insulin release from pancreatic islet β-cells is considered to be one of the causal factors in the etiology of T2DM. Rac1 is particularly important for glucose-stimulated insulin secretion (Wang and Thurmond, 2009). In contrast, RhoA expression is increased in β-cells under diabetic conditions, and Rho/Rho-kinase activation is involved in the suppression of insulin biosynthesis (Nakamura et al., 2006). Thus, insulin release from pancreatic islet β-cells could be determined by the resulting balance between RhoA and Rac1 activities. These findings suggest that Rho GTPase signaling affects not only the dendritic spine structure but also a number of cellular processes, including insulin release from pancreatic islet β-cells, and that aberrations in Rho GTPase signaling, including its activation by GEFs, could therefore contribute to the comorbidity of schizophrenia and T2DM (Figure 1).

### Wnt/β-Catenin

Wnts are secreted glycoproteins known as extracellular ligands. Wnt/β-catenin signaling (canonical pathway) is a critical and well-studied pathway (MacDonald et al., 2009). In the absence of Wnt ligands, cytoplasmic β-catenin protein is tightly regulated at a low level by casein kinase 1 (CK1)-mediated phosphorylation and the regulatory adenomatous polyposis coli (APC)/axin/glycogen synthase kinase-3 (GSK-3β) complex, leading to its ubiquitination and subsequent proteasomal degradation (Gao et al., 2014). In an activated state, Wnt signaling promotes destruction of APC/axin/GSK-3β complex components and inhibition of β-catenin degradation. β-Catenin accumulates in the cytoplasm and eventually translocates into the nucleus, where it binds with T-cell factor/lymphoid enhancer factor (TCF/LEF) family members and induces the transcription of target genes (Shang et al., 2017). The Wnt signaling pathway is crucial for regulating diverse biological processes such as embryonic development, organ formation, and cell proliferation. This pathway plays an important role in the pathophysiology of T2DM and has been shown to be critical for the development of the pancreas and islets during embryonic growth (Papadopoulou and Edlund, 2005). The Wnt/β-catenin pathway also plays a role in neuronal development (Brafman and Willert, 2017).

TCF7L2, also called TCF4, is one of the TCF/LEF family members. The *TCF7L2* gene encodes a high mobility (HMG) box that plays an important role in the downstream Wnt/β-catenin signal pathway (Hansson et al., 2010). Functionally, TCF7L2 is critical for β-cell proliferation and survival as well as insulin production and secretion (Liu and Habener, 2010) and is a key regulator of insulin and proinsulin synthesis and processing (Zhou et al., 2014). Although studies of TCF7L2 in brain development and pathologies have been relatively scarce, evidence from animal studies strongly implicates TCF7L2-dependent transcription in the development of changes in the volume of cortical areas, thalamo-cortical dysconnectivity, and white matter microstructural alterations (Bem et al., 2019). TCF7L2 also regulates synaptic plasticity (Kennedy et al., 2016). Therefore, TCF7L2 may contribute to the comorbidity between schizophrenia and T2DM.

DISC1 also regulates the stability of Wnt/β-catenin signaling by an interaction with GSK3β and acts through this pathway to regulate neural progenitor proliferation and modulate mental homeostasis (Mao et al., 2009). Disheveled-axin domain-containing 1 (DIXDC1), a direct interacting partner of DISC1, contributes to psychiatric pathogenesis by regulating dendritic spine and glutamatergic synapse density downstream of Wnt/β-catenin signaling (Martin et al., 2018).

### Akt/GSK3

GSK3 plays several roles in differentiation and development, intracellular trafficking, apoptosis, and regulation of gene transcription (Emamian, 2012). Some studies suggest that GSK3 in the brain could modulate synaptic plasticity (Emamian, 2012). GSK-3β is a key player of Wnt signaling pathways (Freyberg et al., 2010). GSK3 is a molecule immediately downstream to Akt, a serine/threonine kinase (Freyberg et al., 2010). GSK3 and Akt are serine threonine kinases that were initially identified as playing a role in regulating the activity of glycogen synthesis in response to insulin receptor stimulation (Beaulieu, 2012). Insulin signals through the tyrosine kinase activity of its receptor to activate Akt through the phosphatidylinositol 3-kinase pathway. Akt phosphorylates GSK-3 and inactivates it (Lovestone et al., 2007). Of interest, DISC1 regulates pancreatic beta-cell function, decreases beta-cell proliferation, and promotes apoptosis and glucose intolerance in transgenic mice via regulation of GSK3β (Jurczyk et al., 2016).

GSK3 has also been implicated in the pathogenesis of schizophrenia and the actions of neurotransmission of dopamine (Kaidanovich-Beilin et al., 2012). GSK3 is a major downstream regulator of dopamine receptor D2 (DRD2), which is targeted by most antipsychotics. Besides the canonical G protein-dependent cAMP-protein kinase A signaling pathway, the non-canonical DRD2 transduction pathway is the G protein-independent Akt/GSK3 pathway (Beaulieu et al., 2011). Activation of DRD2 by dopamine facilitates the arrestin 2/protein phosphatase 2A/Akt complex and dephosphorylates and inactivates Akt, followed by dephosphorylation (activation) of GSK3 (Beaulieu et al., 2011). Chronic administration of a dopamine agonist, such as amphetamine or apomorphine, also leads to increased inhibitory phosphorylation of Akt and increased activation of GSK3β (Beaulieu et al., 2011). On the other hand, antipsychotics are able to increase Akt activation (Emamian et al., 2004; Takaki et al., 2018). These data establish a strong relationship between dopamine levels and Akt/GSK3β signaling (Singh, 2013). Akt and GSK-3 may be modulated by DISC1 with indirect and direct interactions, respectively (Dahoun et al., 2017). The Akt/GSK-3 pathway may be responsible for the co-occurrence of T2DM and schizophrenia (Lin and Shuldiner, 2010).

Multiple genetic, functional, and animal studies have shown that COMT is significantly associated with schizophrenia (Luo et al., 2014). COMT is a prime candidate for ameliorating the cognitive dysfunction of schizophrenia (Tunbridge et al., 2006). *COMT* polymorphism is also associated with hyperglycemia and hemoglobin A1C in T2DM (Hall et al., 2016). Furthermore, COMT is related to the Akt/GSK3 cascade. The *COMT* Val108/158Met genotype is related to Akt phosphorylation, and information on functional interactions between COMT and AKt may provide novel insights into the pathogenesis of schizophrenia (Sei et al., 2010).

Increasing evidence reveals regulatory interactions between dopamine and the central renin-angiotensin system (Oh and Fan, 2019). ACE catalyzes the conversion of angiotensin I to the active hypertensive peptide angiotensin II. Angiotensin II induces dopamine release in mesolimbic dopaminergic neurons (Rodriguez et al., 2020). Although several studies reported that ACE activity is inconsistent in patients with schizophrenia, higher ACE activity is associated with cognitive dysfunction in patients with schizophrenia (Rodriguez et al., 2020). Because angiotensin II increases hepatic glucose production and decreases insulin sensitivity, ACE inhibitor and angiotensin receptor blockers are reported to reduce the occurrence of T2DM (Gillespie et al., 2005).

### Common Mechanisms in Schizophrenia and T2DM

**Inflammation Abnormality—**Inflammation is a necessary response to infection, harmful chemicals, and tissue damage (Muller, 2018). Inflammation comes at the cost of a transient decline in tissue function, which in turn contributes to altering the homeostasis and becomes the pathogenesis of diseases (Medzhitov, 2010). The long-term effects of inflammatory mediators induce neuroinflammatory disease in the brain as well as metabolic disease in the pancreas (Bauer and Teixeira, 2019).

The origins of inflammatory and immune activation in schizophrenia include (1) genetic predisposition; (2) prenatal exposure to infections (Brown and Derkits, 2010), maternal inflammation during pregnancy (Canetta et al., 2014), and obstetric complications (Cannon et al., 2002); (3) gastrointestinal permeability and the gut microbiome (Severance et al., 2016); (4) psychological trauma (Popovic et al., 2019; Stilo and Murray, 2019) and other environmental exposures such as a low level of serum vitamin D (Davis et al., 2016) and substance use (Miller et al., 2018); and (5) abnormalities in brain insulin action (Agarwal et al., 2020). These inflammations affect the development and activity of microglial cells, which work in the primary inflammations in the central nervous system (Howes and McCutcheon, 2017). Findings from the current meta-analysis seem to support elevated levels of pro-inflammatory marker cytokines, such as interleukin-6 (IL-6), IL-1β, and tumor necrosis factor A (TNF-A), in the blood and cerebrospinal fluid of patients with schizophrenia (Goldsmith et al., 2016; Capuzzi et al., 2017). Inflammatory markers have been found to be increased in the first episode of schizophrenia, and manifested alterations in the severity and resistance to treatment in various stages of the illness (Fineberg and Ellman, 2013; Upthegrove et al., 2014; Capuzzi et al., 2017). Furthermore, these inflammatory markers are associated with negative symptoms, indicating loss of brain matter and cognitive impairment in patients with schizophrenia (Garcia-Rizo et al., 2012; Fillman et al., 2013; Meyer, 2013).

Low-grade inflammation has also been described as a risk factor for future development of T2DM. Many studies reported increased levels of pro-inflammatory markers, such as IL-6, IL-1β, and TNF-A, which are predictive components in patients with T2DM (Rehman and Akash, 2016). Pediatric studies, which have the advantage of not being influenced by other diseases, medications, or active tobacco smoking, have demonstrated that IL-1β, IL-6, TNF-A, and other markers are increased in insulin resistance (Reinehr, 2019). Thus, pro-inflammatory mediators could promote insulin resistance and β-cell failure, ultimately resulting in the development of T2DM. Psychological stress can be considered to have a significant role in the onset and progression of diabetes (Afrisham et al., 2019). Although the liver and adipose tissue are important sites for the activation of inflammation pathways, chronic stress directly activates the innate immune system, which, in turn, activates the production of IL-6 and other cytokines (Pickup, 2004). With a genetic predisposition, subsequent stress contributes a vulnerability factor for inflammation-associated schizophrenia. Multiple genome-wide association studies have shown that the major histocompatibility complex on chromosome 6p, which is known to play a key role in the immune system, is an important region for allelic association in schizophrenia (Ripke, 2014). In particular, alleles of complement *C4* within the human leukocyte antigen were found to be associated with schizophrenia, and C4 promotes synaptic elimination (Sekar et al., 2016). C4 levels also correlate with body mass index (Copenhaver et al., 2019). *IL-1B*, *IL6*, and *IL6R* genes were associated with schizophrenia in a meta-analysis (Hudson and Miller, 2018) and an association study (Kapelski et al., 2015). Variants in *IL1B*, *IL6*, and other cytokine genes were associated with T2DM in multiple studies (Achyut et al., 2007; Arora et al., 2011; Banerjee and Saxena, 2014). An altered immune system and inflammatory components induced by chronic stress were associated with the molecular mechanisms of diabetes in schizophrenia (van Beveren et al., 2014; Ward and Druss, 2015).

Thus, inflammation may be a common underlying mechanism for schizophrenia and diabetes mellitus, which are highly comorbid with each other (Khandaker et al., 2017).

### Oxidative Stress

Oxidative stress is defined as an imbalance between the production of reactive oxygen species and their elimination by a protective mechanism (antioxidant system), which can lead to chronic inflammation (Hussain et al., 2016). Oxidative stress is harmful because excess reactive oxygen species attacks biological molecules such as proteins and DNA (Yoshikawa and Naito, 2002). An accumulation of oxidative damage to biological molecules is involved in the pathogenesis of various diseases, including metabolic diseases, diabetes complications, and neurodegenerative disorders (Emiliani et al., 2014). Multiple lines of evidence have identified increased oxidative stress in patients with schizophrenia (Koga et al., 2016). Most studies examined markers of oxidative status in the blood, such as endogenous antioxidants glutathione (GSH) (Barron et al., 2017). A recent extensive review found that peripheral markers of GSH were consistently decreased but found equivocal results for other antioxidants such as superoxide dismutase and catalase (Koga et al., 2016). Genetic studies have shown associations between oxidative stress gene polymorphisms and schizophrenia, including genetic variations in a subunit or GSH cysteine ligase, the enzyme responsible for GSH synthesis, and several glutathione S-transferases (GST), utilizing GSH as a co-factor (Gysin et al., 2007; Gravina et al., 2011). Meta-analysis studies have found the association of the most important genes of the GST family, *GSTM1* and *GSTT1* variants, with T2DM (Tang et al., 2013; Zhang et al., 2013a). However, unlike genetic association studies, the available GWASs have not provided convincing evidence for an oxidative stress–related genetic predisposition to schizophrenia (Maas et al., 2017).

*PON1* is a candidate for a gene that overlaps schizophrenia and T2DM. PON1 enzyme is known to have a protective effect against oxidative stress (Menini and Gugliucci, 2014; Bigagli and Lodovici, 2019). In this context, PON1 activity is inversely associated with inflammatory responses. Drug-naïve first-episode patients with schizophrenia show an inverse relationship between decreased activity of the enzyme PON1 and increased cytokine levels, including IL-6, IL-4, and IL-10 (Brinholi et al., 2015). PON1 activity is also decreased in T2DM and related to β-cell function (Meneses et al., 2019).

MTHFR is a key enzyme for 1-carbon metabolism and DNA methylation. *MTHFR* polymorphisms (C677T and A1298C) are related to enzymatic activity, and an approximately 20% reduction of MTHFR enzyme activity is shown in patients with schizophrenia (Wan et al., 2018). Interestingly, these polymorphisms are frequently reported in the onset of T2DM and diabetic nephropathy (Mtiraoui et al., 2007).

### HPA Axis Dysfunction

The HPA axis, a neuroendocrine system, plays a fundamental role in the maintenance of reactions to stress and affects the physiologic adaptive reactions of the organism to stressors (Nicolaides et al., 2015). HPA is involved in the homeostasis of metabolic, cardiovascular, and reproductive systems, as well as the immune system (van den Brink et al., 2018). The central stress system triggers the synthesis and secretion of corticotropin-releasing hormone (CRH) in the paraventricular nuclei of the hypothalamus (Chrousos, 1995). Through the hypophysial portal system, CRH reaches the anterior pituitary gland and releases adrenocorticotropic hormone (ACTH) into the systemic circulation. On binding to the glucocorticoid receptors of the adrenocortical cells, ACTH subsequently induces glucocorticoid synthesis and secretion, which control CRH and ACTH release via a negative feedback loop (van den Brink et al., 2018). Consequently, the blood glucocorticoid concentration is increased by the stress reaction. Glucocorticoids are steroid hormones that regulate multiple aspects of glucose homeostasis. Glucocorticoids promote gluconeogenesis in the liver by induction of gluconeogenesis enzymes and decrease glucose uptake and utilization by antagonizing the insulin response in skeletal muscle and adipose tissue (Chiodini et al., 2007). Therefore, excess or long-lasting (chronic) glucocorticoid exposure causes hyperglycemia and insulin resistance. In patients with T2DM, glucocorticoid secretion has been suggested to be a possible link between insulin resistance and the features of metabolic syndrome (Chiodini et al., 2007).

Epidemiological studies have revealed that HPA activity plays a role in the pathophysiology of schizophrenia. Pro-inflammatory cytokines such as IL-1, IL-6, or TNF are also involved in activating the HPA axis (Chrousos, 1995; Meyer, 2013). Although there are contradictory reports (Ciufolini et al., 2014), schizophrenia is associated with elevated baseline and challenge-induced HPA activity (Walker et al., 2008). Furthermore, control of the HPA axis was also impaired in drug-naïve and first-episode patients with schizophrenia (Ryan et al., 2004), and baseline cortisol levels are higher in prodromal (clinically high risk) patients (Walker et al., 2013). However, these findings are not universal, and there is limited agreement about elevations in glucocorticoids (Bradley and Dinan, 2010).

### Other Endocrine Systems (Prolactin)

The prolactin (PRL) pathway may contribute to the comorbidity of schizophrenia and T2DM (Gragnoli et al., 2016). *PRL* lies on locus 6p22.3, which is strongly associated with T2DM in the GWAS replication study (Lu et al., 2012). PRL plays a role in regulation of beta-cell mass (Nielsen et al., 2001), islet regeneration and proliferation (Nyblom et al., 2009), and insulin secretion (Sorenson and Brelje, 2009). Low PRL levels are related to a higher T2DM risk in both sexes (Balbach et al., 2013). On the other hand, higher PRL levels were associated with lower glucose levels and higher insulin sensitivity (Wagner et al., 2014).

PRL levels are also associated with schizophrenia. First-episode drug-naïve male schizophrenia patients have serum PRL levels 3 times higher than healthy male controls (Albayrak et al., 2014). It has also been shown that the PRL level is negatively associated with the severity of positive psychosis symptoms in drug-naïve male patients with schizophrenia (Ramsey et al., 2013). Furthermore, *PRL* polymorphism is associated with schizophrenia, especially in male patients (Rybakowski et al., 2012). Although increased or decreased PRL levels have not been found consistently across studies or by gender difference (Rajkumar, 2014), PRL dysfunction may sustain disrupted mental development and T2DM-related metabolism.

Other candidate genes, PRL-releasing hormone receptor (*PRLRH*), PRL receptor (*PRLR*), oxytocin (*OXT*), oxytocin receptor (*OXTR*), and *NPY*, may also correlate with the PRL pathway and contribute to schizophrenia and T2DM. However, genetic data on *PRLRH*, *PRLR*, *OXT*, *OXTR*, and *NPY* in human T2DM and schizophrenia patients are scarce (Postolache et al., 2019).

## CONCLUSION

**Figure 2.**
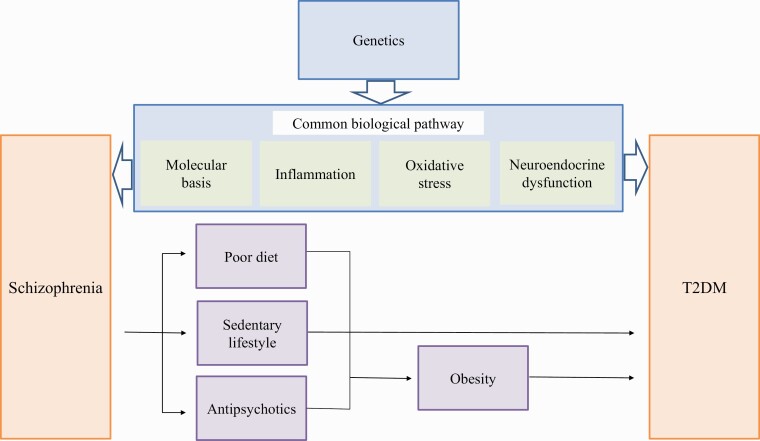
Mechanisms that underlie the association between schizophrenia and type 2 diabetes mellitus (T2DM). The mechanisms of the increasing prevalence of T2DM in patients with schizophrenia are multifactorial. Poor diet and sedentary lifestyle are included in the traditional risk factors. Iatrogenic risk during treatment with antipsychotics is included in risk factors unique to schizophrenia (Ward and Druss, 2015). Accumulating evidence suggests shared genetic susceptibility and biological common pathway of both schizophrenia and T2DM.

We summarized the genetics and functional mechanisms underlying the comorbidity of schizophrenia and T2DM (Figure 2). Even when genetic analyses are performed on a relatively large number of comorbid patients, the results are sometimes inconsistent, and susceptibility genes may also have only a low or moderate risk to the onset of both. Genetic association studies have revealed the number of common risk variants underlying diseases, but these variants explain only a proportion of heritability. Among the reasons for the complexity in this field are suspected to be the following: (1) the heterogeneity of schizophrenia; (2) many environmental factors, such as lifestyle and vulnerability to life events, which are related to genetic factors; and (3) both genetic and environmental factors that affect common mechanisms in schizophrenia and T2DM, such as abnormal inflammation, oxidative stress, and HPA axis dysfunction. It is very difficult to distinguish purely environmental factors from purely genetic factors. A new approach is to estimate environmental factors statistically compensated by digitized intermediate phenotypes directly related to genetic factors, such as cognitive function, structural or functional magnetic resonance imaging analysis, and cerebral blood flow.

On the other hand, based on many genetic and epidemiological studies, the comorbidity of schizophrenia and T2DM is established, and it is probably safe to assume that common cascades and mechanisms suspected from common genes’ functions in the brain or pancreas are related to the onset of schizophrenia and T2DM. At the point of preemptive medicine, genetic and epidemiological information will be used in making decisions for the prevention and treatment of schizophrenia and T2DM. Though introduction of new medications or supplements that are effective in these cascades and mechanisms may be expected, 1 target point may not be adequate because each common cascade and mechanism is also closely linked. In the future, in addition to more comprehensive whole genome and epigenome analyses of schizophrenia and T2DM, a more precise approach such as familial and rare gene analyses of CNVs of comorbid patients, further subdivision of the diagnoses of schizophrenia, and the following basic functional research may eliminate these difficulties and clarify the treatment target for patients with the same phenotype but different causes.

## Interest Statement

Y.Y. has received honoraria for speaking at educational events sponsored by Novartis and Dainippon Sumitomo. N.Y. has received unrestricted research funding from Daiichi Sankyo, Eisai, Pfizer, Otsuka, Astellas, and Merck Sharp & Dohme, which was deposited into research accounts at Okayama University. N.Y. has also received honoraria for his participation as a speaker at educational events from UCB Japan, Tsumura, Pfizer, Dainippon-Sumitomo, Daiichi-Sankyo, Merck Sharp & Dohme, Pfizer, Eisai, Meiji-Seika, and Mochida. M.T. has received honoraria for his participation as a speaker at educational events sponsored by Daiichi Sankyo, Takeda, Tsumura, Otsuka, and Dainippon Sumitomo. S.S. has received unrestricted research funding from Eli Lilly, which was deposited into research accounts at Okayama University Hospital. S.S. has received honoraria for his participation as a speaker at an educational event sponsored by Otsuka and Meiji-Seika. Y.M. has received honoraria for his participation as a speaker at educational events sponsored by Otsuka and Dainippon Sumitomo. N.H. received honoraria for his participation as a speaker at educational events sponsored by Otsuka, Eisai, and Nippon Shinyaku. Y.O. reports no additional financial or other relationship relevant to this article.
